# DNA Damage and Survival Time Course of Deinococcal Cell Pellets During 3 Years of Exposure to Outer Space

**DOI:** 10.3389/fmicb.2020.02050

**Published:** 2020-08-26

**Authors:** Yuko Kawaguchi, Mio Shibuya, Iori Kinoshita, Jun Yatabe, Issay Narumi, Hiromi Shibata, Risako Hayashi, Daisuke Fujiwara, Yuka Murano, Hirofumi Hashimoto, Eiichi Imai, Satoshi Kodaira, Yukio Uchihori, Kazumichi Nakagawa, Hajime Mita, Shin-ichi Yokobori, Akihiko Yamagishi

**Affiliations:** ^1^ School of Life Sciences, Tokyo University of Pharmacy and Life Sciences, Hachioji, Japan; ^2^ Faculty of Life Sciences, Toyo University, Oura-gun, Japan; ^3^ The Institute of Scientific and Industrial Research, Osaka University, Ibaraki, Japan; ^4^ Institute of Space and Astronautical Science, Japan Aerospace Exploration Agency (JAXA), Sagamihara, Japan; ^5^ Department of Bioengineering, Nagaoka University of Technology, Nagaoka, Japan; ^6^ National Institute of Radiological Sciences, National Institutes for Quantum and Radiological Science and Technology, Chiba, Japan; ^7^ Department of Life, Environment and Applied Chemistry, Faculty of Engineering, Fukuoka Institute of Technology, Fukuoka, Japan

**Keywords:** panspermia, cell-aggregate, *Deinococcus* spp., international space station, exposure facility

## Abstract

The hypothesis called “panspermia” proposes an interplanetary transfer of life. Experiments have exposed extremophilic organisms to outer space to test microbe survivability and the panspermia hypothesis. Microbes inside shielding material with sufficient thickness to protect them from UV-irradiation can survive in space. This process has been called “lithopanspermia,” meaning rocky panspermia. We previously proposed sub-millimeter cell pellets (aggregates) could survive in the harsh space environment based on an on-ground laboratory experiment. To test our hypothesis, we placed dried cell pellets of the radioresistant bacteria *Deinococcus* spp. in aluminum plate wells in exposure panels attached to the outside of the International Space Station (ISS). We exposed microbial cell pellets with different thickness to space environments. The results indicated the importance of the aggregated form of cells for surviving in harsh space environment. We also analyzed the samples exposed to space from 1 to 3 years. The experimental design enabled us to get and extrapolate the survival time course to predict the survival time of *Deinococcus radiodurans*. Dried deinococcal cell pellets of 500 μm thickness were alive after 3 years of space exposure and repaired DNA damage at cultivation. Thus, cell pellets 1 mm in diameter have sufficient protection from UV and are estimated to endure the space environment for 2–8 years, extrapolating the survival curve and considering the illumination efficiency of the space experiment. Comparison of the survival of different DNA repair-deficient mutants suggested that cell aggregates exposed in space for 3 years suffered DNA damage, which is most efficiently repaired by the *uvrA* gene and *uvdE* gene products, which are responsible for nucleotide excision repair and UV-damage excision repair. Collectively, these results support the possibility of microbial cell aggregates (pellets) as an ark for interplanetary transfer of microbes within several years.

## Introduction

Panspermia hypothesis postulates that microscopic forms of life, such as spores, can be dispersed in interplanetary space and thereby seed life from one planet to another (Arrhenius, 1908). Experiments have exposed extremophilic organisms to outer space to test microbe survivability and the panspermia hypothesis (Horneck et al., 2010; Cottin et al., 2017). Multilayers of *Bacillus subtilis* spores under space conditions with UV-irradiation beneath a perforated aluminum dome survived up to 6 years in the space mission of Spacelab and long duration exposure facility (LDEF), although single layer spores were killed (Horneck 1993, Horneck et al., 1994). However, no further analyses on the time course of survival, effect of spore thickness, effect of mutations, and DNA damage were completed. Microbes inside shielding material (e.g., small fragments of rock and mixtures of sugar or clay) with sufficient thickness to protect them from UV-irradiation can survive in space (Horneck et al., 2001; Onofri et al., 2012; Bryce et al., 2015; Panitz et al., 2015). This process has been called “lithopanspermia,” meaning rocky panspermia (Melosh, 1988; Mileikowsky et al., 2000; Horneck et al., 2002; Nicholson, 2009; Worth et al., 2013).

Terrestrial microbes have been isolated from air samples collected in the troposphere and stratosphere, and because they were detected using cultivation methods, these captured microbes must have been protected from UV. The microbes may be physically protected from UV by shielding and/or may have the appropriate molecular mechanisms to deal with UV-induced damage. Some microbes isolated at high altitude tend to form clumps or cell aggregates (Lighthart, 1997; Harris et al., 2002; Wainwright et al., 2004), which may have provided UV protection. For example, we previously isolated *Deinococcus aerius* and *Deinococcus aetherius*, two new species of the genus *Deinococcus*, from air dust collected at the upper troposphere and low stratosphere, respectively (Yang et al., 2008, 2009a, 2010). Deinococcal colonies can easily grow larger than 1 mm in diameter. Our previous on-ground laboratory experiment showed that deinococcal cells near the surface layer of aggregates are killed by UV rays, but the layers of killed cells protect the cells underneath from UV damage (Kawaguchi et al., 2013). Sub-millimeter cell aggregates (pellets) of *Deinococcus radiodurans*, *D. aerius*, and *D. aetherius* would be expected to survive the low Earth orbit environment, including exposure to vacuum, temperature changes, heavy ions, and γ-rays, for 1 year (Kawaguchi et al., 2013). We previously proposed that these cell aggregates might act as an ark for interplanetary transfer of microbes, and named the concept “massapanspermia,” where *massa* means mass in Latin (Kawaguchi et al., 2013).

To investigate the concept of the massapanspermia in space, we performed exposure experiments of dried deinococcal cell pellets at the Exposure Facility of the Japanese Experimental Module (JEM) of the International Space Station (ISS) orbiting about 400 km above the Earth’s surface (Kawaguchi et al., 2016; Yamagishi et al., 2018), during the space mission “Tanpopo,” which means dandelion in Japanese.

We exposed the microbial cell pellet with different thickness to space environments. The results indicated the importance of the aggregated form of cells for surviving in harsh space environment. We also analyzed the samples exposed to space from 1 to 3 years. The experimental design enabled us to get and to extrapolate the survival time course and to predict the survival time of *D. radiodurans*. The results supported the concept of the massapanspermia if other requirements are met, such as ejection from the donor planet, transfer, and landing.

## Materials and Methods

### Experimental Set up

**Figure 1:**
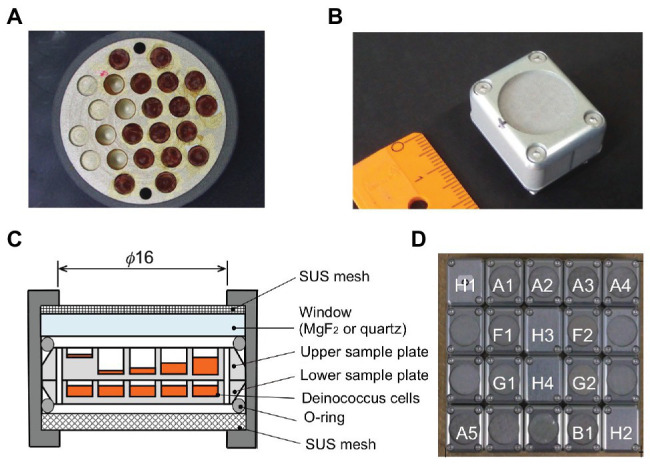
Experimental tools in the Tanpopo mission. **(A)** Sample plate (18 mm in diameter) with wells (2 mm in diameter) filled with deinococcal cells. Image **(B)** and cross-section **(C)** of an exposure unit. A metal mesh was placed at the top of the window to prevent scattering of accidentally broken windows. Wells of the upper sample plate were filled with deinococcal cells to different depths. The lower sample plate contained the dark control samples (modified from Kawaguchi et al., 2016). **(D)** Each exposure panel was comprised of 20 exposure units (modified from Kawaguchi et al., 2016). A1–A4: *D. radioduras* wild type R1 and mutant strains KH311, rec30, and UVS78, under MgF_2_ window. A5: *D. aerius* TR0125 under MgF_2_ window. B1: *D. radioduras* R1 under quartz window. F1 and F2: Alanine VUV dosimeter under MgF_2_ window. G1 and G2: Alanine VUV dosimeter under SiO_2_ window. The windows of F2 and G2 were coated with Au neutral density filter. H1, H2, H3, and H4: Ionization radiation dosimeter.

Wells of 2.0-mm diameter on the aluminum plate were filled with different amounts of dehydrated cells of *D. radiodurans* and *D. aerius*, as well as mutants of *D. radiodurans* deficient in DNA repair genes (Kawaguchi et al., 2016; Figure 1A). Two aluminum plates with bacterial samples were stacked inside each exposure unit (Figures 1B,C). Twenty exposure units were arranged in each exposure panel (EP), as shown in our previous report (Yamagishi et al., 2018; Figure 1D). During the mission, three EPs were exposed for different durations from 1 to 3 years.

### Bacterial Strains, Culture, and Sample Preparation

*Deinococcus radiodurans* strain R1 ATCC 13939 was cultured for 15 h in mTGE medium [1% (w/v) Bacto tryptone, 0.6% (w/v) beef extract, and 0.2% (w/v) glucose] at 30°C in an incubator with continuous shaking at 150 rpm until it reached late logarithmic phase. *D. aerius* strain TR0125 JCM 11750 was cultured for about 4 days in mTGE medium at 30°C with shaking at 150 rpm. The following DNA repair-deficient mutants were also used: *D. radiodurans* KH311 (deficient in *pprA* gene), *D. radiodurans* rec30 (deficient in *recA* gene), and *D. radiodurans* UVS78 (deficient in *uvrA* and *uvdE* genes). Strains KH311, UVS78, and rec30 were cultured in mTGE medium at 30°C with shaking at 150 rpm for between 48 and 72 h.

Cells of *D. radiodurans* R1 and *D. aerius* were harvested by centrifugation at 5,000 rpm, 4°C for 10 min and washed three times and resuspended with 10 mM potassium phosphate buffer (PB; pH7.0). Sterilized aluminum plates with cylindrical wells (2.0 mm diameter and 2 mm or 100 μm depth) with a flat floor were used as sample holders (Figure 1A). The cell suspension was dropped into the wells and dried under 3.3 × 10^−2^ atm in a desiccator (SANYO, SPD−WVGS300) on a clean bench. The cell suspension dropping and drying steps were repeated about 15–20 times, with the final drying step conducted for more than 16 h under 3.3 × 10^−2^ atm in a desiccator. The wells were filled with different amounts of deinococcal cells corresponding approximately to a 1-μm thick single layer of cells, and 100-, 500-, 1,000-, and 1,500-μm-thick cell layers, in the upper sample plate (Figure 1C). Three *D. radiodurans* mutant strains were also placed in the upper sample plates to about 1, 500, 1,000, and 1,500 μm thickness. The wells were filled with dried cells at 1,000 μm thickness in the lower sample plates (Figure 1C), and in the ISS cabin and ground control sample plates.

To achieve the designated thickness, the required volume of cell suspension was determined from the cell concentration estimated from optical density at 590 nm of cell culture as described previously (Kawaguchi et al., 2013), and the cell number required to achieve the 2,000 μm thickness determined in pre-experiment. The calculated volume of cell suspension was applied to the wells.

Dried cell pellets corresponding to 1,000 or 1,500-μm thickness were picked up from wells and the pellet heights were measured using a microscope (OLYMPUS SZX7, Tokyo, Japan) equipped with a CCD camera (OLYMPUS DP73, Tokyo, Japan). Examples of the photo images of a *D. radiodurans* R1 cell pellet removed from the well of the ground control plate and the plate exposed to space under an MgF_2_ window are shown in Figure 1.

The actual cell numbers were estimated by colony counting of the cell suspension used for sample preparation and are shown in Supplementary Table 2. The actual thickness of 1,000 or 1,500 μm space exposed sample was measured under the microscope as described above, then was used to estimate the actual thicknesses of the other samples, combining it with the volumes of the cell suspension used for preparation. The actual thicknesses of the samples are shown in Supplementary Table 1.

An upper sample plate and a lower sample plate were stacked in an exposure unit (Figure 1C). The upper sample plates in the exposure units would be UV-irradiated, whereas the lower sample plates would be non-UV-irradiated and act as a dark control. All the upper sample plates were placed under the MgF_2_ window, except one *D. raiodurans* R1 sample plate, which was placed under quartz window (Figure 1D). For the ISS cabin control, EPs were packed in zippered plastic bags with two desiccant blocks each and kept in the dark in the pressurized storeroom of JEM-ISS. Ground control samples were stored in an incubator with desiccant blocks at 20°C in our laboratory at Tokyo University of Pharmacy and Life Sciences, Japan.

### Payload and Experimental Conditions of the Tanpopo Mission

The Tanpopo EPs were attached to the Exposure Handrail Attachment Mechanism (ExHAM) and placed on the Exposure Facility of the JEM-ISS, as described in the references (Kawaguchi et al., 2016; Yamagishi et al., 2018). All biological samples were prepared from October 2014 to February 2015. Biological samples and UV and cosmic radiation dosimeters were assembled on one EP (Figure 1D). EPs were packed in plastic bags with desiccant blocks during transportation. On 14 April 2015, 20:10:41 UTC, EPs with other space samples were launched on board Space-X CRS-6. Three EPs were mounted on the top face of the ExHAM by Scott Kelly (NASA’s astronaut). To avoid the possible effect of degassing from the sample to the window, EPs were exposed to space vacuum for 12 days in an airlock of JEM. The ExHAM was attached to the Exposure Facility of JEM-ISS by a robotic arm. The same type of EP was stored in the ISS pressurized storage area (ISS storage room JPM1O2_c2) as an ISS cabin control. After 384 days of exposure, the ExHAM was retrieved into the ISS by a robotic arm on 13 June 2016 (UTC). The first-year EP was detached from the ExHAM and packed in a plastic bag with desiccant blocks. The first-year EP and ISS cabin control were returned to Earth, landing in the Pacific Ocean, *via* SpaceX-9 on 27 August (UTC), and were returned to us in September 2016.

The ExHAM was returned to the same position on the Exposure Facility of JEM-ISS by a robotic arm. After a total of 769 days of exposure, the second-year EP was detached from the ExHAM and stored in the ISS pressurized area. The second-year EP and ISS cabin control were returned to Earth *via* Space X12 on 17 September 2017 (UTC), and returned to us in October 2017.

The ExHAM was returned to the same position on the Exposure Facility of JEM-ISS by a robotic arm. After a total of 1,126 days of exposure, the third-year EP was detached from the ExHAM and stored in the ISS pressurized area. The third-year EP and ISS cabin control were returned to Earth *via* Space X15 on 2 August 2018 (UTC), and returned to us in August 2018.

We estimated the UV flux with an alanine film dosimeter as described previously (Yamagishi et al., 2018). Alanine film was formed by vacuum sublimation technique on an MgF_2_ plate, coated with hexatriacontane, and mounted in Exposure Units. After 1-, 2-, and 3-year exposure in the space environment, degradation of alanine was evaluated by infrared absorbance measurement at 1,307 cm^−1^. We determined UV absorption dose between 120 and 203 nm with the calibration curve that was obtained by measuring the alanine degradation vs. 172 nm UV irradiation dose using Xe_2_ excimer lamp.

Aluminum oxide-based optically stimulated luminescence dosimeters (Yamagishi et al., 2018) and silver-activated phosphate glass-based radiophotoluminescence dosimeters were used to measure radiation dosimetry outside and inside the ISS (Yamagishi et al., 2018). Temperature was monitored by a mechanical thermometer (Yamagishi et al., 2018). Environmental conditions are summarized in Supplementary Table 3.

### Survival Assay

After exposure, the dehydrated cells were recovered from the sample plate wells by resuspending the cell pellet in 0.5 ml sterile PB for each well, and used for analyses. Aliquots of deinococcal cell suspension were serially diluted in sterile PB and dropped onto mTGE medium plates. Colonies were counted after incubation at 30°C for 36 h (*D. radiodurans* R1), 2 days (*D. radiodurans* KH311and UVS78), 4 days (*D. radiodurans* rec30), and 3 days (*D. aerius*). Surviving cell fractions were determined from the quotient *N*/*N*_0_, where *N* was the number of colony-forming units of the sample kept in the space, ground, or ISS cabin, and *N*_0_ was that at the time of sample preparation.

Recovered cells from three wells were separately used for the slope and Y-intercept analysis of the surviving fraction, for each thickness, condition, strain, and year. Coefficient of determination (*R* ^2^), *p*, and *t* values was estimated to evaluate the regression line. The *t* test of difference of slopes of regression lines and differences of Y-intercept of regression lines were performed according to Ichikawa (1990). In the case of no correction for multiple comparisons, *p* < 0.05 was judged to reject null hypotheses [for slope, no difference between slopes of two regression lines (*H*0), for Y-intercept, no difference between Y-intercepts of two regression lines (*H*0)]. As correction method of multiple comparisons, Benjamini and Hochberg (1995) correction method was used.

### Quantitative PCR

From the recovered cell suspension 0.5 ml, we used the volume corresponding to 1.0 × 10^7^ cells and 1.0 × 10^8^ cells of original cell count at the time of space sample preparation, to prepare DNA from the 100-μm thick and 500‐ or 1,000-μm thick samples, respectively. The cells were collected by centrifugation at 14,000 rpm, 4°C for 10 min, and used for DNA preparation using the DNeasy Blood and Tissue Kit (QIAGEN, Hilden, Germany) according to the manufacturer’s manual. Genomic DNA was dissolved in 100 μl of T_10_E_0.1_ (10 mM Tris-HCl, 0.1 mM EDTA, pH 8.0) (Affymetrix, Cleveland, United States). The concentration of the extracted DNA solution was measured by an absorption spectrometer using absorbance at 260 nm (U-2910, HITACHI, Tokyo, Japan). DNA was also extracted from the freshly harvested *D. radiodurans* R1 to obtain the standard curve for quantitative PCR (qPCR).

For qPCR, DNA templates were amplified with KOD SYBR qPCR Mix (TOYOBO, Osaka, Japan) in accordance with the manufacturer’s recommended procedure. Oligonucleotide primers (Eurofins Genomics, Tokyo, Japan) for the RNA polymerase *β* subunit (*rpoB*) gene in *D. radiodurans* R1 were used (forward, 5'-AAA CTG TGC CGA TGG AC-3', 5' nucleotide at position 1,058; and reverse, 5'-TAG CTC ACG CGG CCA TTC AC-3', 5' nucleotide at position 1945). Primers and templates were dissolved in T_10_E_0.1_ (Affymetrix). Each 30-μl qPCR reaction contained 15 μl of PCR reaction buffer, 0.1 μm each forward and reverse primers, 1 μl ROX reference dye and 1 μl of extracted genomic DNA. The reactions were carried out in 0.2-ml clear PCR tubes in an Applied Biosystems StepOne Real-Time PCR System (Thermo Fisher Scientific, Waltham, United States) under the following conditions: 98°C for 2 min, and then 35 cycles of 98°C for 10 s, 60°C for 10 s, and 68°C for 1 min with fluorescence signal measurement. Threshold cycle (C_t_) values were determined from each sample and converted to the number of copies of the *rpoB* gene of *D. radiodurans* by using a standard curve prepared from serial dilutions of template DNA prepared from freshly harvested cells. The *rpoB* gene copy number of the standard DNA was estimated from the DNA concentration and the genome size. Measurements were conducted in triplicate on serial 100-fold dilutions of DNA solution. For all samples, the amplification efficiency was at least R^2^ = 0.992. To confirm that the expected PCR product was produced, melting points were determined at the end of each qPCR assay.

### Pulsed-Field Gel Electrophoresis

Cell suspensions recovered from the 1,000-μm thickness samples in the 1-year upper and lower sample plates were used for pulsed-field gel electrophoresis (PFGE) analysis. Sixty microliter aliquots containing 8.0 × 10^6^ *D. radiodurans* cells (calculated from the initial cell number of dried cells when prepared the space sample) was mixed with 60 μl of 3% molten agarose (Certified Low Melt Agarose, Bio-Rad Laboratories, Hercules, United States) in Multibuffer (10 mM Tris, 40 mM EDTA, 50 mM sucrose, 0.1% Triton X-100, and pH 8.0) and solidified in the well of a disposable plug mold (Bio-Rad) to obtain a gel plug. The plug was incubated with 0.5 ml of 1 mg/ml lysozyme buffer in Multibuffer for 24 h at 37°C with gentle agitation, followed by 1 mg/ml of proteinase K in Multibuffer at 55°C for 48 h with gentle agitation. The plug was washed with 2 ml of sterile ultrapure water and then with TE buffer (10 mM Tris-HCl, 1 mM EDTA, and pH 8.0).

A 2.5-mm gel slice cut from the plug with a twin edge razor blade (containing 2.0 × 10^6^ cells) was digested with 10 units of *Not*I (New England Biolabs, Ipswich, United States) in 100 μl of buffer at 37°C overnight. The slice was then washed and placed at the forefront of the plastic comb of the PFGE gel casting stand and subjected to PFGE using a Bio-Rad CHEF Mapper XA, with 1% agarose (Bio-Rad Megabase Agarose) in 0.5x TBE (44.5 mM Tris, 44.5 mM boric acid, and 1 mM EDTA) and the following conditions: gradient 4.0 V/cm, run time 18 h, included angle 120°, initial switch time 10 s, final switch time 90 s. Gels were stained with 1 μg/ml of ethidium bromide, gel images were captured and viewed using a fluorescent imaging system (BioTools DigiPrint Doc Tablet DP-T130z, Maebashi, Japan). The cropping of the gel images was carried out using Preview on macOS High Sierra (Apple, Cupertino, CA, United States) to draw electrophoregram. Raw gel image files before cropping are shown in Supplementary Figure 4. A range between bands of 259 and 479 kb was selected as a region of interest (ROI). Contrasts of the ROIs were adjusted, and their intensity values were measured by using ImageJ (Rasband, W.S., ImageJ, U. S. National Institutes of Health, Bethesda, Maryland, United States, http://imagej.nih.gov/ij/, 1997–2016). The results were compared statistically by one-way analysis of variance (ANOVA) with *post hoc* Tukey’s honestly significant difference (HSD) test using software SPSS Statistics (version 26, IBM). Different letters above the columns indicate statistically significant differences between groups (*α* = 0.05).

## Results

### Surviving Fraction of Wild-Type *Deinococcus* spp.

The *D. radiodurans* R1 cell pellets were removed from the aluminum plate wells. There was no apparent deterioration of the texture or structure in the space sample compared with the ground control, except that the color of the space samples changed from red to slightly yellowish red (Supplementary Figure 1). This difference might be caused by UV irradiation.

**Figure 2:**
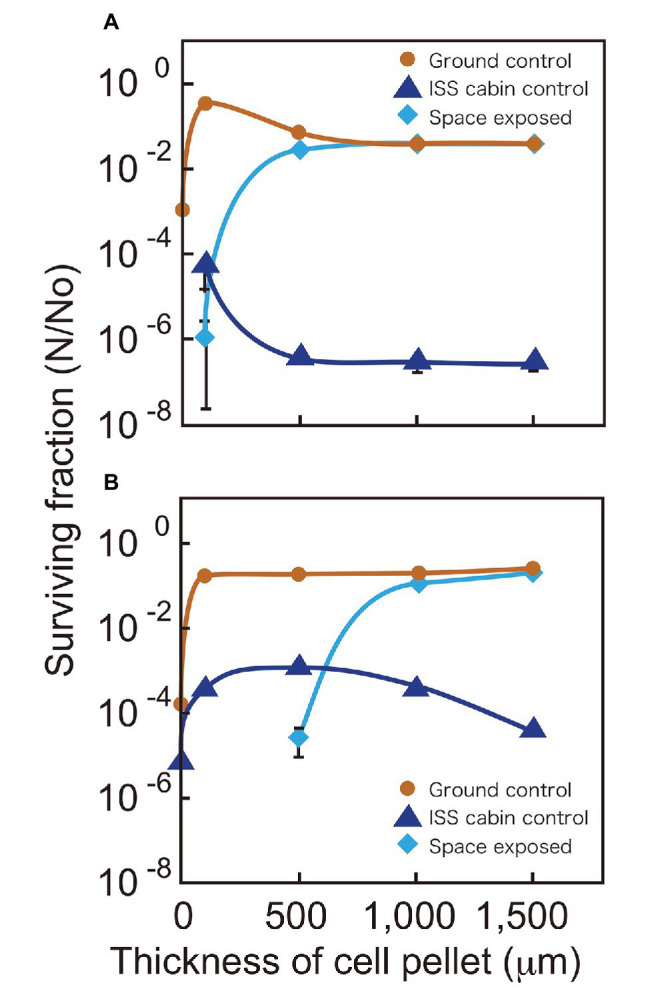
Surviving fractions of *D. radiodurans* R1 **(A)** and *D. aerius* TR0125 **(B)** after 3 years. Cells were under a MgF_2_ window (UV >110 nm) when exposed to space (pale blue diamonds). The International Space Station (ISS) cabin (blue triangles) and the ground controls (brown circles) are also shown. Far left data on the vertical axis show the results of sample with about 1 μm thickness. Some data points (1 μm ISS cabin control and space exposed samples in A and 1 and 100 μm space exposed samples in B) are not indicated because no surviving cells were detected. The actual thicknesses of the samples are presented in Supplementary Table 1. Each error bar shows the standard error of the mean (SEM) of triplicate samples; most SEMs were small and covered by data markers.

We compared the surviving fractions of cell pellets kept in space, the ISS pressurized area (cabin control), and the ground laboratory for 3 years (Figure 2). The ground controls of *D. radiodurans* R1 survived for 3 years irrespective of cell pellet thickness. The ISS cabin controls showed reduced survival compared to the ground controls after 3 years of exposure (Figure 2A), this result will be discussed later.

Although the 100-μm thick space samples of *D. radiodurans* R1 exposed to UV showed low survival, cell pellets with a thickness of 500 μm or greater showed survival similar to that of the ground controls (Figure 2A). Another deinococcal species, *D. aerius*, showed similar survival rates but with different thickness dependence (Figure 2B). The 100-μm thick space samples of *D. aerius* exposed to UV did not survive, pellets of 500 μm thickness had low survival, but those of 1,000 μm thickness and greater showed survival rates similar to that of the ground control. Our previous study showed that the extinction coefficients *α* (μm^−1^), representing the efficiency in decreasing the UV intensity depending on the depth of cell layer, at VUV172 nm and UVC254 nm are higher for dried *D. radiodurans* R1 cells than for dried *D. aerius* cells (Kawaguchi et al., 2013). The previous data support the survival patterns observed here. The difference between *D. radiodurans* R1 and *D. aerius* strains may result from variation in the extinction coefficients of the strains.

### Time Course of Survival Fractions of Wild-Type *D. radiodurans* R1

**Figure 3:**
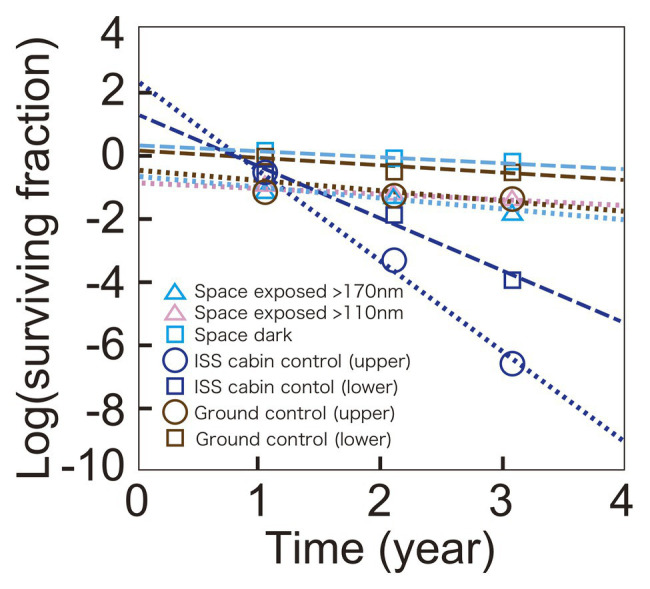
Time course of survival of *D. radiodurans* R1 exposed to space at UV >170 nm (pale blue triangles) or UV >110 nm (pink triangles), or kept in the dark (pale blue squares). The ISS cabin upper (blue circles), lower (blue squares), and the ground upper (brown circles) and lower (brown squares) controls are also shown. Data of upper plate samples are connected by dotted lines. Data of lower plate samples are shown in squares and connected by dashed lines. The data of 1,000-μm thick samples are shown. The actual thicknesses of the samples are presented in Supplementary Table 1. Each mark shows the mean of triplicate samples; SEM was small and covered by data markers.

The time course of the surviving fraction of *D. radiodurans* R1 cells with 1,000 μm thickness are shown in Figure 3. Each sample showed a logarithmic decrease in survival over 3 years. Slopes were similar between samples of ground control, space exposed, and space exposed dark samples but Y-intercepts were different between these samples. However, the difference may be at least partially related to the lot of the sample preparation. Samples for the same location but different durations were prepared as one lot. For example, all the upper sample plates of *D. radiodurans* R1 (space exposed, ISS cabin control and ground control) for 1-, 2-, and 3-year were prepared as one lot. While, all the lower plates of *D. radiodurans* R1 for 1-, 2-, and 3-year were prepared as another lot, suggesting the possible difference between the upper-plate‐ and the lower-plate-samples. The surviving fraction of samples in the same lot decreased similarly during the time in storage before starting the space experiments. Therefore, the slope of the time course may be more reliable than Y-intercept. For example, the difference in the Y-intercepts between the upper and lower sample plates of ground control samples may be due to variation between sample lots or slight differences in humidity.

Survival of the cabin control samples decreased faster than other samples, and mortality rate accelerated, resulting in a positive Y-intercept. The cabin control samples were stored in plastic bags with desiccant blocks. The humidity of the bags may have increased causing the accelerating mortality rate in the presence of atmospheric oxygen in ISS cabin. Note that very low oxygen and humidity are expected for space exposed samples.

### Expected Survival Time of Wild-Type *D. radiodurans* R1

The survival times of *D. radiodurans* R1 were estimated from the survival curves and the initial cell numbers and are listed in Table 1. Expected survival time with the *R* ^2^ (coefficient of determination; Supplementary Table 5) higher than 0.7 are shown in bold letter. The cell pellets with thickness greater than 0.5 mm were expected to survive in space between 15 and 45 years exposed to UV and 48 years in the dark. However, the ISS Exposure Facility is not always exposed to sunlight; ISS was behind the Earth every 90 min and EPs were shaded by Solar Array Wings or the ISS body depending on the direction and configuration of the ISS.

**Table 1:** Survival time estimated from the survival time course of *Deinococcus radiodurans* R1.

Space exposure condition	Thickness (μm)	Survival time^*^ (year)	Expected survival time range in interplanetary space^**^ (year)
MgF_2_	500	35.6 ± 0.5	4.3–6.1
	1,000	43.4 ± 0.2^***^	5.2–7.5
	1,500	45.3 ± 0.5	5.5–7.8
SiO_2_	500	14.8 ± 0.5^***^	1.8–2.6
	1,000	24.6 ± 0.7	3.0–4.2
	1,500	30.8 ± 0.7	3.7–5.3
Dark	1,000	48.1 ± 0.1^***^	48.1 ± 0.1

* Survival time with 95% probability was estimated from the survival time course and initial cell numbers (Supplementary Table 2).

** Expected survival time range was estimated from the survival time and the UV dose of the experiments (between 44 and 63 equivalent solar day (ESD)/year under MgF_2_ window and between 41 and 58 ESD/year under quartz window in interplanetary space, Supplementary Table 4).

*** Expected survival time with the *R* ^2^ (coefficient of determination) higher than 0.7 (Supplementary Table 5) is shown in bold letters.

The UV illumination efficiency was monitored using a passive integral alanine film dosimeter (Yamagishi et al., 2018). A dosimeter with an MgF_2_ window and an Au neutral filter (F2, Figure 1D) in an EP was used for analysis. The alanine films in two dosimeters without Au neutral filters (F1 and G1) were denatured too much to estimate the dose. The film with a quartz window and a neutral filter (G2) needs more detail analysis of the optical transparency in VUV region. The absorbance of F2 alanine filters at 1,307 cm^−1^ decreased from 0.34 before the space experiment to 0.30 after the space experiment, from 0.38 to 0.28 and from 0.28 to 0.173 in the first-, second-, and third-year alanine film, respectively. The absorption data were used to estimate the VUV dose. Time course of VUV dose between 120 and 203 nm is shown in Supplementary Figure 2A. Each data set was estimated from respective standard curve obtained from one alanine standard film covered with hexatriacontane (Supplementary Figure 2B). Hexatriacontane coating was used to protect alanine film from vacuum. However, the alanine film showed unexpected variation in dose dependent denaturation curve. The origin of the variation is not clear yet, though it may be related to the variation in the thickness of the hexatriacontane coating, which is not easily evaluated. Average UV flux was estimated to be between 470 and 670 kJm^−2^ yr^−1^ in front of the exposure unit between 120 nm to 203 nm, derived from three standard curves. The values correspond to from 11 to 16% of the solar irradiance *I*_sun_ between 120 and 203 nm, which is 4.07 MJm^−2^ yr^−1^ (Lean, 1991). VUV dose between 120 and 203 nm in front of microbe sample under the MgF_2_ window was between 120 and 170 kJm^−2^ yr^−1^ (Supplementary Table 4). The value corresponds to from 3.0 to 4.2% of the solar irradiance *I*_sun_ between 120 and 203 nm.

When considering the microbe pellet floating interplanetary space, pellet is expected to rotate around the axis whose direction is randomly changed by dust collision. If we assume a sphere with radius *r* that is rotating around randomly flipping axis in the interplanetary space, the sunlight energy entering the cross section of a particle *πr* ^2^ is distributed over the total surface area 4*πr* ^2^. Thus *I*_sun_/4 is expected to be the total energy per unit area on the sphere. Using these values with the transparency of the mesh and windows at VUV region, UV dose under the MgF_2_ window is equivalent to between 44 and 63 ESD/year, where ESD stand for the equivalent solar day in the interplanetary space, and the UV dose under the quartz window was between 41 and 58 ESD/year (Supplementary Table 4). Considering the efficiency of the UV illumination in the EP, expected survival is from 2 to 8 years with UV irradiation, and 48.1 ± 0.1 years without UV exposure (Table 1).

Mean absorbed dose of ionization radiation as a function of mission year from 2015 to 2018 were estimated (Supplementary Figure 3). Total absorbed doses during 3-year mission were 715 ± 15 mGy (SD) in space and 256 ± 2 mGy (SD) in pressurized area of ISS. The annual dose rates were 232 ± 5 mGy/year (SD) and 83 ± 1 mGy/year (SD) in space and pressurized area of ISS, respectively. The dose is too low to affect the survival of *D. radiodurans* (Kawaguchi et al., 2013).

### Survivability of *D. radiodurans* DNA Repair-Deficient Mutants

Protection of DNA is essential for survival in space. Several types of DNA damage are caused by environmental factors in space, including pyrimidine dimerization induced by solar UV (Horneck, et al., 1984; Onofri et al., 2012), double-strand breaks (DSBs) generated by high-dose ionizing radiation (Kobayashi et al., 1994; Sikorsky et al., 2004; Moeller et al., 2012) or desiccation (Yang et al., 2009b, Bauermeister et al., 2011), single-strand breaks (SSBs; Dose et al., 1992), and base deletion and insertion induced by high vacuum (Moeller et al., 2010).

We have investigated DNA damage caused by exposure in space in the wild-type and mutant *D. radiodurans* cells as a function of the thickness of the cell pellets. *D. radiodurans* strain KH311 is deficient in condensed nucleoid-dependent end joining (CNDEJ), owing to a mutation in *pprA* gene (Kitayama et al., 1983; Narumi et al., 2004; Ishino and Narumi, 2015). *D. radiodurans* strain rec30 is deficient in extended synthesis-dependent strand annealing (ESDSA) and homologous recombination (HR), owing to a mutation in *recA* gene (Moseley and Copland, 1975; Narumi et al., 1999). In addition, *D. radiodurans* strain UVS78 is deficient in nucleotide excision repair (NER) and UV-damage excision repair (UVER), owing to mutations in *uvrA* and *uvdE* genes (Moseley and Evans, 1983; Narumi et al., 1997; Kitayama et al., 2003).

**Figure 4:**
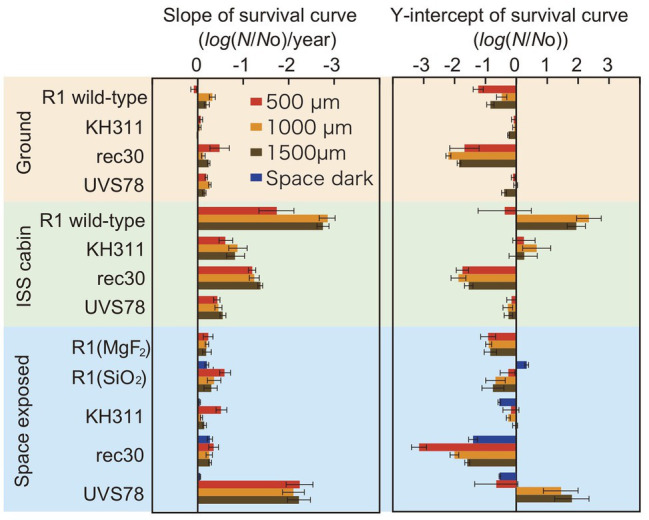
Slope and Y-intercept of the survival curve of pellets of *D. radiodurans* wild type and DNA repair-deficient mutants at different thickness exposed to space from 1 to 3 years. Samples were either stored at the ground laboratory (top pale brown box) or ISS cabin (middle pale green box) or exposed to space (bottom pale blue box) under MgF_2_ windows (UV >110 nm). *D. radiodurans* R1 was exposed also under SiO_2_ windows (UV >170 nm) Red, orange and brown bars: 500-, 1,000-, and 1,500-μm thick samples. Blue bars: exposed to the space environment without UV irradiation. Each error bar shows the SEM. See also Supplementary Tables 5, 6 for the statistical analysis data.

To assess the type of damage that most significantly affects survival in space, we compared the slope and Y-intercept of the survival curves of *D. radiodurans* mutants exposed to space, stored at the ground laboratory or in the ISS cabin (Figure 4). The statistical analysis data are shown in Supplementary Tables 5, 6. The slopes of the survival curves of the ground control samples were similar between wild type and mutants, with some variation depending on the thickness. The slopes of the ISS cabin controls were steeper than the ground controls for each strain.

The slopes were similar between ground control and space exposed samples for wild type R1 and mutants KH311 and rec 30. This result suggests that no significant DNA damage occurred due to space environment factors that would be repaired by ESDSA and HR facilitated by the *recA* gene (Moseley and Copland, 1975; Narumi et al., 1999) and by CNDEJ facilitated by the *pprA* gene (Kitayama et al., 1983; Narumi et al., 2004; Ishino and Narumi, 2015). The slope of space exposed samples of strain UVS78 was steeper than the other strains and space dark control of strain UVS78 (Supplementary Table 6), suggesting that short-wavelength UV-induced damage is more frequently repaired by the *uvrA* gene and *uvdE* gene products through NER and UVER (Moseley and Evans, 1983; Narumi et al., 1997; Kitayama et al., 2003), than by ESDSA/HR and CNDEJ. UV-induced DNA damage, mainly pyrimidine dimerization, was caused by short-wavelength UV, and is most effectively repaired by the *uvrA* gene and *uvdE* gene products. These genes are most important for survival in space with UV exposure.

The Y-intercepts of survival curves of the strains are shown in Figure 4. *D. radiodurans* rec30 had a negative value irrespective of the conditions (Supplementary Table 5). The same process under different conditions, such as during preparation or recovery of the sample, may cause damage repaired by ESDSA/HR.

### DNA Damage Estimated by q-PCR

**Figure 5:**
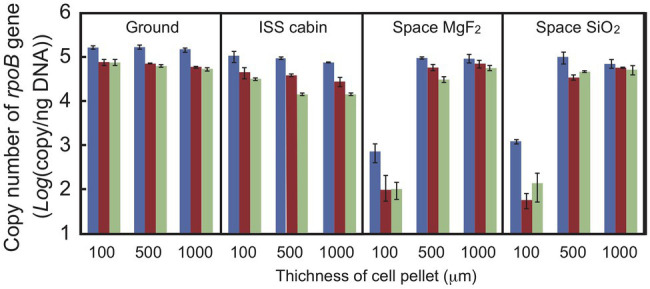
Copy number of the intact *rpoB* gene in DNA prepared from *D. radiodurans* R1 cell pellets of different thicknesses. The intact *rpoB* gene (887 bp) in genomic DNA was amplified and quantified by quantitative PCR (qPCR). Blue, brown and pale green bars represent 1-, 2-, and 3-year exposed samples, respectively. Each error bar shows the SEM of triplicate samples.

Quantitative PCR was used to estimate the DNA damage in a short region of the gene (Sikorsky et al., 2004; Fajardo-Cavazos et al., 2010). Copy numbers of the intact *rpoB* gene in an 887-bp region were estimated by qPCR using total genomic DNA extracted from dehydrated cells of *D. radiodurans* R1 (Figure 5). Copy number was used as an estimate of DNA damage because the DNA polymerase reaction will stall at strand breaks and damaged bases in the amplified region. The number of intact copies of the *rpoB* gene in genomic DNA extracted from freshly harvested cultures was quantified as 3.5 × 10^5^ copies/ng by qPCR. The amount of intact *rpoB* gene copies in the ground and ISS cabin controls after 1 year was only slightly lower than this value. For cell pellets at 100 μm thickness, the amount of intact *rpoB* gene fragment in space UV-irradiated for 1-year samples (>110 and >170 nm) was 100-fold lower than in the ground and ISS cabin controls (*p*-value < 0.001; Figure 5), and even less in 2‐ and 3-year samples. Intact *rpoB* gene copy decreased by only 10-fold in 500-μm thick and 1,000-μm thick space exposed cell pellets compared to ground control (Figure 5). These results support the higher survival of *D. radiodurans* R1 in thicker pellets after exposure to UV-irradiation in space (Figure 2).

Copy number of intact *rpoB* gene decreased in second‐ and third-year samples stored in the ISS cabin (Figure 5). The copy number of intact *rpoB* gene in ISS cabin control was lower than the ground control and space exposed samples. The results might be related to the accelerated mortality rate of ISS cabin samples (Figures 3, 4). However, the slope of each survival curve of DNA repair-deficient mutant stored in ISS cabin was shallower than that of the wild type strain (Figure 4). If the DNA damage occurred in ISS cabin control sample would be related to the accelerating mortality rate, the slopes of mutants would have been steeper than the wild type. Accordingly, the mechanism underling the accelerating mortality rate of the samples stored in ISS cabin is not clear yet.

### Double Strand Breaks Caused by Environmental Factors in Space

**Figure 6:**
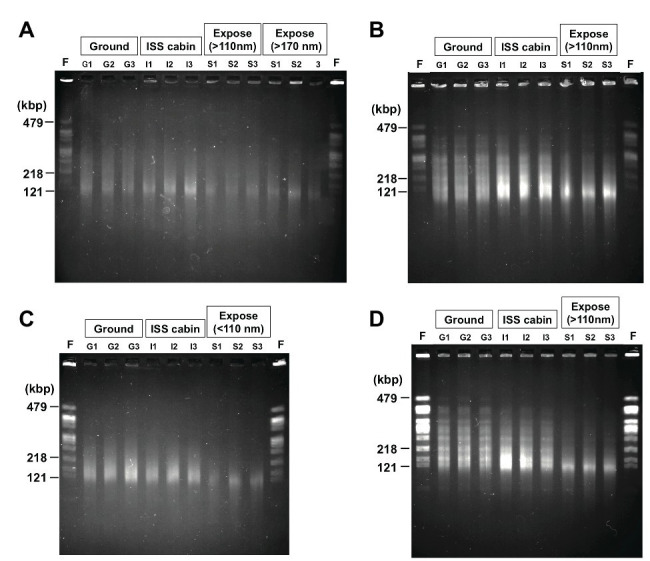
Photo images of the pulsed field gel electrophoresis of the DNA from *D. radiodurans* R1 **(A)**, UVS78 **(B)**, rec30 **(C)**, and KH311 **(D)** prepared from 2.0 × 10^6^ cells of the 1-year samples. *Not*I digested genomic DNA recovered from three different wells of aluminum plates in each condition were analyzed (Lanes G1, G2, G3 from ground controls; lanes I1, I2, I3 from ISS cabin controls; lanes S1, S2, S3 from space exposed samples). Lane F was *Not*I fragments of genomic chromosomal DNA prepared from freshly cultured *D. radiodurans* R1 2.0 × 10^6^ cells. The fragment sizes (479, 218, and 121 kbp) of freshly cultured *D. radiodurans* R1 cells are indicated. The raw gel image files before cropping are shown in Supplementary Figure 4.

Radiation is known to induce DSBs in *D. radiodurans* genomic DNA, resulting in fragmentation (Zimmermann et al., 1994; Battista et al., 1999). DSBs are also induced by extreme desiccation (Yang et al., 2009b). The ionizing radiation doses expected for the ground control, ISS pressurized area, and space environments are shown in Supplementary Table 3. We estimated the proportion of DSBs in genomic DNA prepared from deinococcal cells by using PFGE (Figure 6).

Digestion of the genome of freshly cultured *D. radiodurans* with the restriction enzyme *Not*I yields several separable fragments (Figure 6) as reported by Kikuchi et al. (1999). Although the same number (2.0 × 10^6^ cells) of cells was used for each lane in PFGE, the intensities of the *Not*I fragments appeared in freshly prepared sample and of the fragments recovered from the 1,000-μm samples (G1, G2, G3 for ground controls; I1, I2, I3 for ISS cabin; S1, S2, S3 for space exposed samples) were different (Figure 6). The intensity of the fragments from wild type R1 (Figure 6A) was lower than those of other DNA repair-deficient mutant strains examined (Figures 6B–D). This is an unexpected result for us. This difference may be due to the difference of ploidy level among these strains. It has been shown that *D. radiodurans* is a polyploid bacterium which possesses 4–10 genome copies per cell (Hansen, 1978). The DNA repair-deficient mutant strains KH311, UV78, and rec30 used in this study may possess increased genome copies compared to wild type R1 in order to make up for their lack of DNA repair capacity.

Compared to the freshly prepared sample, fragment pattern became unclear and smeared even in the ground and ISS cabin controls kept for 1 year. This result indicated that a large amount of DSBs occurred in the genome of dried *D. radiodurans* cells after 1 year. The humidity in the ground and ISS cabin controls may promote the production of DSBs. Despite this, *D. radiodurans* showed a considerably high survival rate in 1,000-μm ground and space exposed samples after 3 years (Figure 2A). These results highlight the extraordinarily high DSB repair ability of this bacterium. In almost all cases, the amount of DSBs in space exposed sample significantly increased compared to those of ground and ISS cabin controls regardless of UV irradiation (Supplementary Figure 4). This result suggests that space environmental factors (Supplementary Table 3) other than UV fluence induced additional DSBs in the dried *D. radiodurans* cells. The survival curve of space exposed wild type R1 after 3 years (Figure 2A) emphasizes again the extremely high DSB repair ability of *D. radiodurans*.

## Discussion

The ISS cabin controls of *D. radiodurans* R1 showed reduced survival compared to the ground controls after 3 years of exposure (Figure 2A). This may be attributed to differences in humidity between the two environments, among other factors. The environmental conditions are summarized in Supplementary Table 3. Humidity in the ISS cabin and on ground controls were around 45–50 and 5–15%, respectively (Supplementary Table 3). Cells inside ISS cabin samples could not be kept dry during the experimental period and this moisture may have caused oxidative stress. Oxygen partial pressure in the ISS cabin did not differ from ground control. The slopes of the ISS cabin controls were steeper than the ground controls also for each strain of DNA deficient mutant (Figure 4 and Supplementary Table 6). This result may be also related to the environmental factors, such as the relatively higher humidity in the ISS cabin, or other unknown factors (Supplementary Table 3).

To test the effect of solar VUV between 110 and 170 nm, the UV with high photon energy, we have exposed and compared the survival of *D. radiodurans* R1 cells under MgF_2_ and quartz windows. The slopes of the samples are summarized in Figure 4. No significant difference of the slopes was noted between samples with different windows, when the sample thickness is 1,000 or 1,500 μm (Supplementary Table 6). The VUV between 110 and 170 nm is less than 1% of sun light energy between 110 and 400 nm (Supplementary Table 4) and may not be harmful to *D. radiodurans* R1 cell pellet with the thickness 1,000-μm or more. Slope of the sample with 500 μm thickness showed steeper slope under the quartz window than the MgF_2_ window, despite the lower total UV energy under the quartz window (Supplementary Table 4). However, the reason is not clear yet.

In the space experiment ADAPT, bacterial endospores of the highly UV-resistant *B. subtilis* strain MW01 were exposed to low-Earth orbit (LEO) for 559 days on board the European Space Agency’s exposure facility EXPOSE-E, mounted outside the ISS (Wassmann et al., 2012). They reported that if shielded from solar irradiation, about 8% of MW01 spores survived in LEO conditions, compared to the laboratory controls. Their results demonstrated the effect of shielding against the high inactivation potential of extraterrestrial solar UV radiation, which limits the chances of survival of even the highly UV-resistant strain of *B. subtilis* MW01 in the harsh environments of outer space.

In our space experiment, though the 100-μm thick cell pellets of *D. radiodurans* R1 and *D. aerius* TR0125 barely survived pellets of 500 μm thickness or greater survived exposure in space for 3 years (Figure 2). DNA was heavily damaged by UV in the 100-μm thick pellets of *D. radiodurans*, resulting in a low copy number of the intact *rpoB* gene (Figure 5). The surface color of cell pellets exposed to space changed slightly (Supplementary Figure 1). UV irradiation might have bleached the cells by cleaving carbon bonds in deinococcal carotenoids (Horneck et al., 2001). Photochemical discoloration was also observed at the surface of *Bacillus* spores in a previous space experiment (Horneck et al., 2012). By contrast, there was no detectable change in the color of the middle or bottom parts of the cell pellet (Supplementary Figure 1). The fraction of surviving cells and the amount of accumulated DNA damage were similar in cell pellets of 500 μm thickness or 1,000 μm thickness. These results suggest a shielding effect provided by the surface layer of dead cells that sufficiently protected the cells underneath from UV.

During exposure in space, if the cell pellet was irradiated with UV from only one direction as in the ISS exposure experiment, then the dark side is always protected from UV. However, if the cell pellet would be irradiated with UV from all directions during the process of transferring through interplanetary space, then the center of the cell pellet needs to be protected from UV from all directions. The diameter needed to protect UV from all directions is roughly twice as large as the depth needed to protect UV from one direction. We propose that sub-millimeter cell pellets would be sufficient to protect the internal cells from intense UV irradiation in space.

In previous space exposure experiments of microbes, each exposure experiment was performed independently for only one time period. In the Tanpopo mission, by contrast, experiments with different exposure periods at the same place were conducted. Thus, we can plot the survival fractions after 1, 2, and 3 years of exposure to obtain the time course. The slope and Y-intercept of the time course can be used to separate the time-dependent effect and the effect before and after exposure (or storage).

By analyzing the time course, it is also possible to estimate survival for longer periods (Figure 3). The cell pellets with a thickness greater than 0.5 mm are expected to survive between 15 and 45 years of exposure to UV on ISS EF and 48 years exposure to space in the dark (Table 1). Considering the efficiency of the UV illumination on the EP, from 40 to 60 ESD per year, expected survival is estimated to be from 2 to 8 years in interplanetary space.

Although the flight time of meteoroids traveling between Mars and Earth is in the range of 10^7^ years, the flight time may be only a few months to years, though the frequency of the shortest time travel is very low (Mileikowsky et al., 2000; Frösler et al., 2017). Accordingly, Deinococcal cell pellets in the sub-millimeter range would be sufficient to allow survival during an interplanetary journey from Earth to Mars or Mars to Earth. Cell pellets of 1,000 μm diameter would be able to survive the shortest travel time in space.

The current space experiments were done under the condition with UV >110 nm, temperature fluctuation, ionization radiation, and space vacuum, which are listed in Supplementary Table 3. Most of the environmental factors are similar to those encountered in interplanetary space except UV. The UV dose has been calibrated to match those encountered in interplanetary space in Table 1. Either MgF_2_ or quartz window was used in our experiments. The window may have protective effect to ionization radiation and atomic oxygen. The ionization radiation was monitored under the same protection as the MgF_2_ or quartz window by adjusting the areal density in front of the ionization radiation dosimeter (0.6 g/cm^2^) in our experiment. In the previous report (Yamagishi et al., 2018), we have analyzed the ionization radiation dose depending on the shielding areal density, and found that estimated ionization radiation without window is not larger by 20% of the dose estimated in this report. Atomic oxygen is present in LEO. However, the atomic oxygen is much less in interplanetary space. Accordingly, the surviving time estimates obtained here are the best estimates so far obtained in space experiments. However, the experiment outside the Van Allen belt may give us a chance to obtain better estimates of the surviving time in interplanetary space in future.

Current work provided the surviving time estimates of cell pellets exposed to space (from 2 to 8 years) and in rocks (several tens of years). The values are useful to estimate the frequency of panspermia processes. Provability of panspermia processes may be evaluated by combining the surviving time estimates with the provability of other processes, such as ejection from the donor planet, transfer and landing. It is also important to note that the estimates can be applied to the organism sufficiently evolved to have DNA repair system to be resistant against space environments.

## Data Availability Statement

All datasets presented in this study are included in the article/Supplementary Material.

## Author Contributions

YK, SY, IN, and AY designed the research. HH contributed to the design and manufacture of EPs and contributed as an operator representing the Tanpopo team. YK, MS, IK, JY, RH, DF, and YM analyzed the survival fractions. JY and IN performed qPCR and PFGE, respectively. SK, YU, KN, EI, HS, HM, and HH analyzed the environmental data. YK, SY, IN, HS, and AY wrote the paper. All authors contributed to the article and approved the submitted version.

### Conflict of Interest

The authors declare that the research was conducted in the absence of any commercial or financial relationships that could be construed as a potential conflict of interest.
