# Supplementary material for: DNA Damage and Survival Time Course of Deinococcal Cell Pellets During 3 Years of Exposure to Outer Space
Source: Front Microbiol. 2020 Aug 26;11:2050. doi: 10.3389/fmicb.2020.02050 (PMC7479814; doi:10.3389/fmicb.2020.02050)
Supplement: Supplementary file 1 [file Data_Sheet_1.PDF]

Supplementary Material

1 Supplementary Figures and Tables

1.1 Supplementary Figure

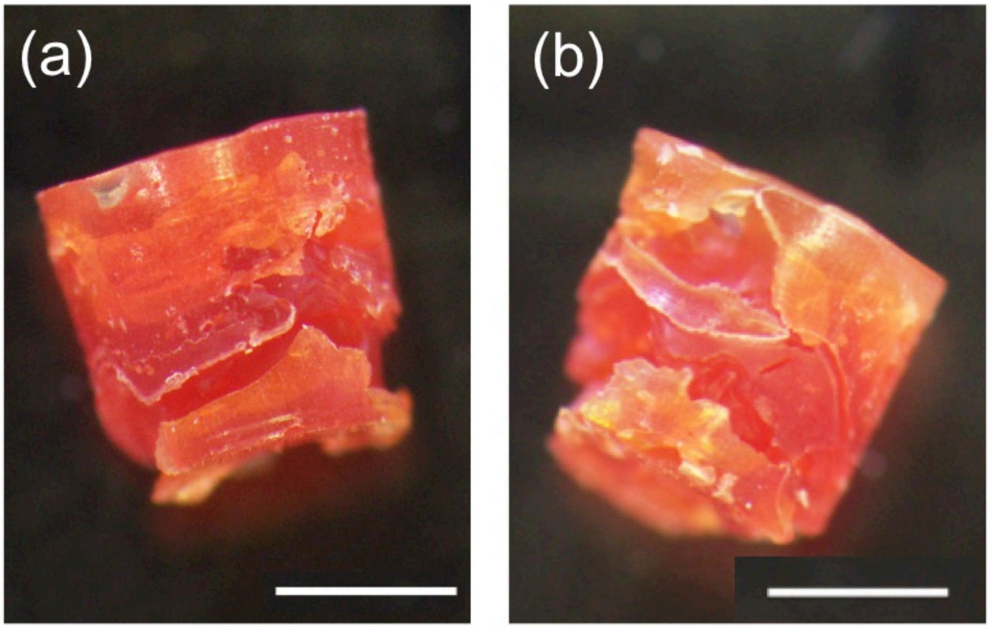

Supplementary Figure 1. *D. radiodurans* cell aggregates (1000 µm thick; 1-year samples). (a) Ground control sample, and (b) sample exposed to UV irradiation in space (under the MgF_2 window). UV irradiated the top layer of cells (b). The cell aggregates were removed from the wells of aluminum plates. The lower parts of the cell aggregates were partially broken during the removal process in some cases. White bars show 1.0 mm.

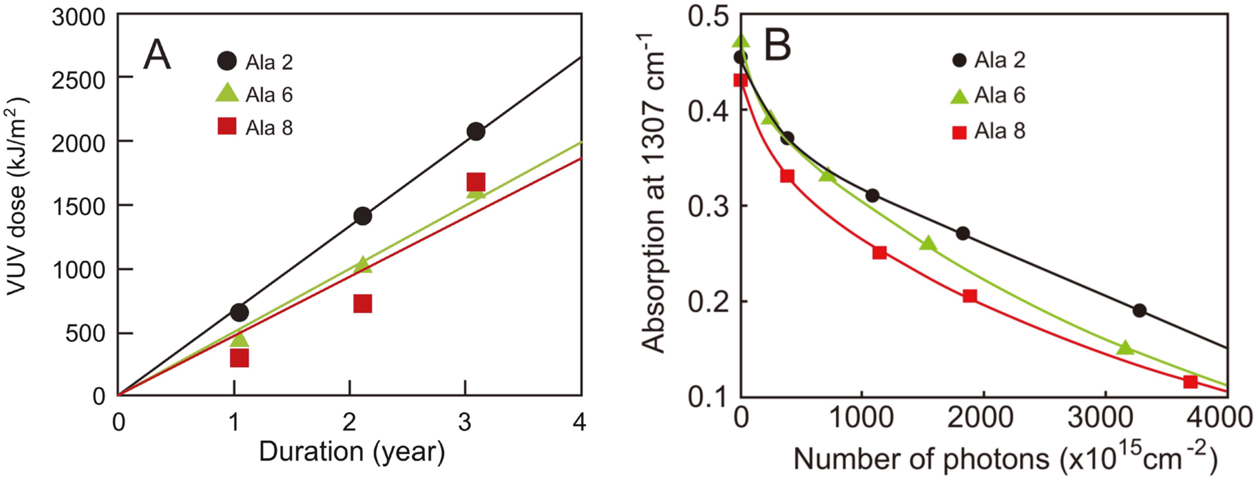

Supplementary Figure 2. UV dose during the space experiment obtained with an alanine dosimeter.

A. Time course of the VUV dose obtained by space exposed alanine dosimeter estimated from Supplementary Figure 2B and the absorbance data: The absorbance at 1307 cm^{-1} decreased from 0.34 before the space experiment to 0.30 after the space experiment, from 0.38 to 0.28 and from 0.28 to 0.173 in the first-, second- and third-year alanine film, respectively. Black circles, green triangles and red rectangles are the data obtained from the standard curve Ala2, Ala6 and Ala8 in Figure 2B, respectively.
B. Standard curves of the alanine film dosimeter. Alanine content of the film was estimated by monitoring the absorbance at 1307 cm^{-1} after the UV irradiation using Xe_2 excimer lamp in the vacuum chamber of the ground laboratory. Black circles, green triangles and red rectangles are data of Ala2, Ala6 and Ala8 alanine film, respectively.

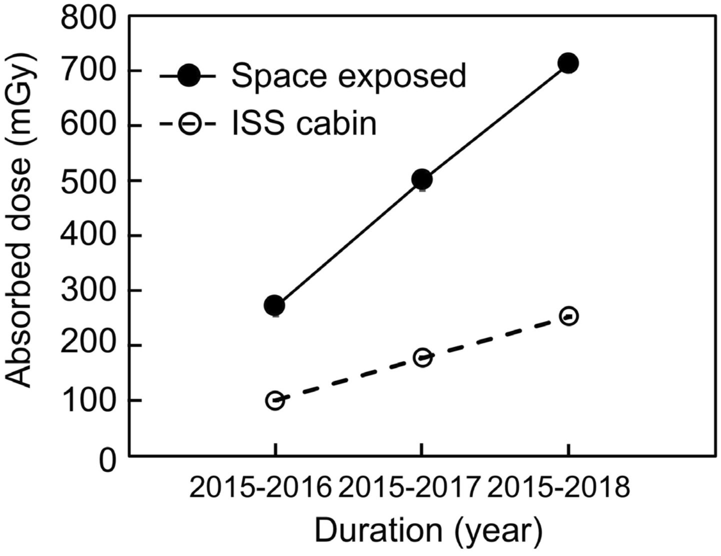

Supplementary Figure 3. Absorbed ionization radiation dose during the space experiment. Error bars indicate the standard deviation. Most of the error bars are small and covered by marks or a line.

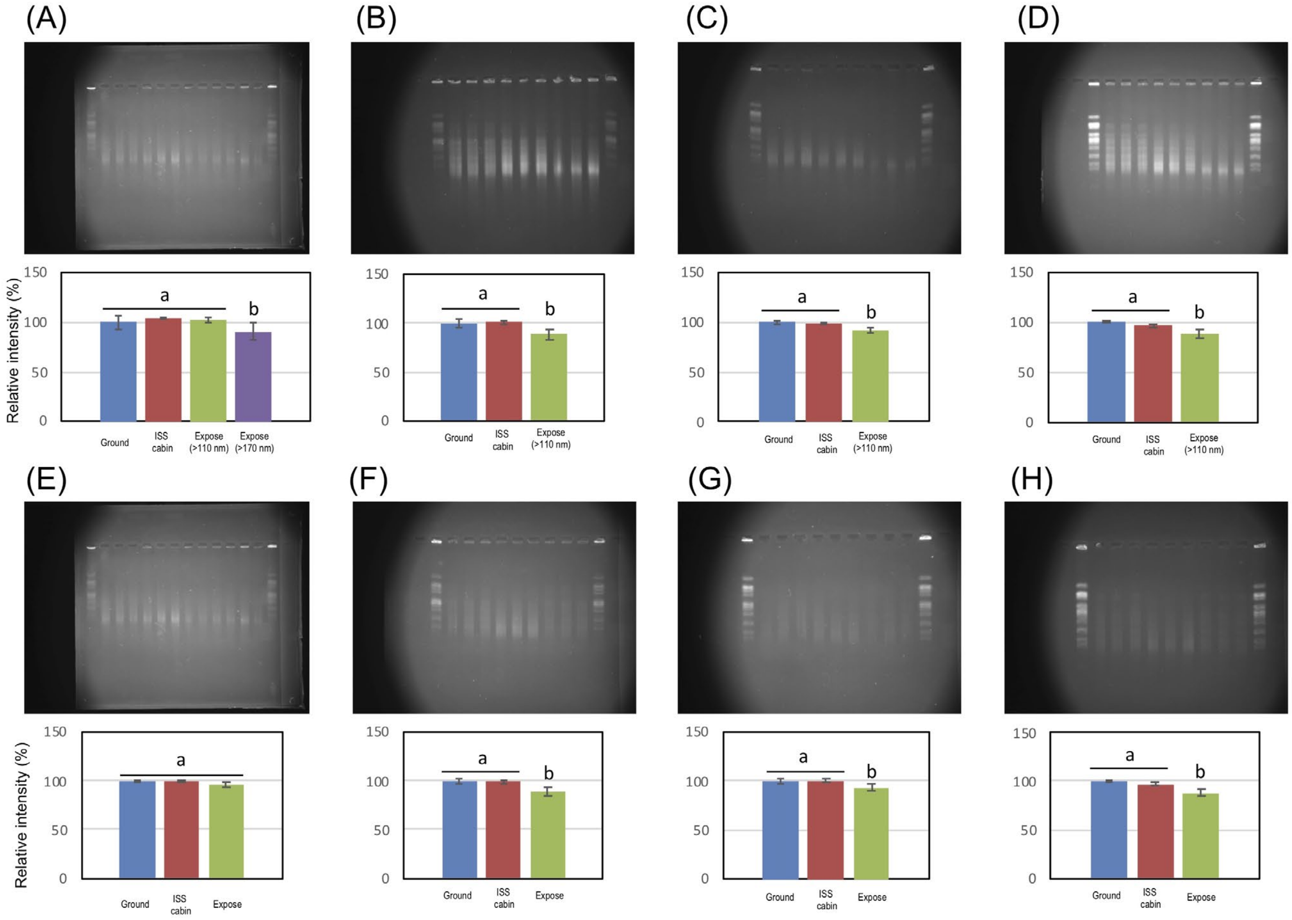

Supplementary Figure 4. Original raw images of PFGE shown in Fig. 6; *D. radiodurans* R1 (A), KH311 (B), UVS78 (C), rec30 (D), and the dark control; R1(E), KH311 (F), UVS78 (G), rec30 (H). Relative intensity of the ROI measured according to Materials and Methods is shown under each photo. The order of lanes in (E), (F), (G) and (H) are the same as that of (B) in Fig. 6. Each error bar shows the SEM of triplicate samples. Different letters above the columns indicate statistically significant differences between groups calculated by one-way ANOVA with Tukey's post-hoc HSD test.

1.2 Supplementary Table

Supplementary Table 1.
Actual cell thickness of* Deinococcus *spp. filled in wells of aluminum plates

	Cell thickness (μm)				
Thickness stated in text<br>Species	1	100	500	1000	1500
D. radiodurans R1	1	113	564	1128	1692
D. radiodurans KH311	1	−	600	1201	1801
D. radiodurans UVS78	1	−	548	1097	1645
D. radiodurans rec30	1	−	619	1238	1857
D. aerius TR0125	1	110	560	1100	1700

Supplementary Table 2.
Total number of deinococcal cells filled in wells of aluminum plates according to cell thickness

	Total number of deinococcal cells				
Thickness stated in text<br>Species	1 μm	100 μm	500 μm	1000 μm	1500 μm
D. radiodurans R1	2.4×10^6	2.0×10^8	9.8×10^8	1.5×10^9	1.6×10^9
D. radiodurans KH311	2.2×10^6	−	1.8×10^8	3.5×10^8	4.8×10^8
D. radiodurans UVS78	3.6×10^8	−	3.6×10^8	7.0×10^8	1.1×10^9
D. radiodurans rec30	2.6×10^6	−	2.7×10^8	5.2×10^8	7.8×10^8

Supplementary Table 3.
Environmental factors in Tanpopo mission during 3-year exposure experiment

Experimental place	Wavelength range (nm) /window type	UV fluence[*a] ($MJ/m^2/year$)	Ionizing radiation (mGy/year)[*b]	Temperature range (°C)[*c]	Pressure range (Pa)[*d]	Humidity (%)
Space						
Upper plate[*e]	110–400/ MgF_2	124 – 177	232 ± 5	29 ± 5 ~ –42 ± 5	10^{-4} ~10^{-7}	–
Upper plate[*e]	170–400/ SiO_2	114 – 163	232 ± 5	29 ± 5 ~ –42 ± 5	10^{-4} ~10^{-7}	–
Dark control in space						
Lower plate[*f]		–	232 ± 5	29 ± 5 ~ –42 ± 5	10^{-4} ~10^{-7}	–
ISS pressurized area		–	83 ± 1	19~25	10^5	45~55
Ground control[*g]		–	1	20	10^5	5~15

*a: UV fluence was calculated using the data from the UV dosimeter as shown in Supplementary Fig. 2 and listed in Supplementary Table 4.
*b: Ionizing radiation was measured with the dosimeters as shown in Supplementary Fig. 3.
*c: Maximum and minimum temperatures were measured as in the reference (Yamagishi et al. 2018).
*d: Outside pressure estimated in Rabbow et al. (2015).
*e: Sample plates were set on the upper side of the exposure unit; the plates were irradiated with UV.
*f: Sample plates were set under the upper plates; UV was completely blocked by the upper plates.
*g: The ground control was stored in a desiccator in an incubator at Tokyo University of Pharmacy and Life Sciences.

Supplementary Table 4.
UV fluence of the sunlight and the values estimated in this work.

Wave length range (nm)	UV fluence ($MJm^{-2}y^{-1}$ or ESD in parentheses)			
	Sunlight	EP surface	MgF_2 window	SiO_2 window
120-203	4.07*	0.470 – 0.670	0.120 – 0.170	
110-400	4086*	469 – 669	124 – 177 (44 – 63)	
170-400	4086*	469 – 669		114 – 163 (41 – 58)

* The values of the sunlight fluence were estimated from the reference (Lean 1991).

Supplementary Table 5

Coefficient of determination (R^2), slope and Y-intercept of regression line of the survival data of* Deinococcus radiodurans *wild type R1 and DNA-repair-gene-mutant strains.

Exposure conditions				Regression coefficient (= slope)				Y-intercept			
Strains	Thickness of exposed cell aggregates (μm)	Coefficient of determination (R^2)	Adjusted R^2	Regression coefficient	SE of regression coefficient	*t* value	*p* value	Y-intercept	SE of Y-intercept	*t* value	*p* value
Ground											
R1	500	0.124	-0.001	0.069	0.070	0.994	0.353	-1.254	0.156	-8.018	**8.98E-05**
	1000	**0.760**	**0.725**	-0.325	0.069	-4.704	**0.002**	-0.494	0.155	-3.194	**0.015**
	1500	0.660	0.612	-0.199	0.054	-3.688	**0.008**	-0.846	0.121	-7.005	**2.11E-04**
KH311	500	0.223	0.112	-0.055	0.039	-1.416	0.200	-0.081	0.087	-0.927	0.385
	1000	0.180	0.063	-0.035	0.028	-1.238	0.255	-0.078	0.063	-1.242	0.254
	1500	0.470	0.395	0.021	0.008	2.492	**0.041**	-0.285	0.019	-15.176	**1.30E-06**
rec30	500	0.404	0.318	-0.465	0.213	-2.176	0.066	-1.662	0.478	-3.474	**0.010**
	1000	0.567	0.505	-0.108	0.036	-3.027	**0.019**	-2.209	0.080	-27.693	**2.06E-08**
	1500	**0.879**	**0.862**	-0.225	0.031	-7.141	**1.87E-04**	-1.853	0.071	-26.270	**2.96E-08**
UVS78	500	**0.915**	**0.903**	-0.182	0.021	-8.699	**5.32E-05**	-0.106	0.047	-2.267	0.058
	1000	**0.916**	**0.904**	-0.260	0.030	-8.730	**5.20E-05**	-0.038	0.067	-0.575	0.583
	1500	**0.725**	**0.686**	-0.138	0.032	-4.300	**0.004**	-0.393	0.072	-5.461	**0.001**
ISS cabin											
R1	500	**0.744**	**0.708**	-1.744	0.386	-4.513	**0.003**	-0.378	0.866	-0.437	0.675
	1000	**0.974**	**0.970**	-2.855	0.176	-16.191	**8.34E-07**	2.331	0.395	5.900	**0.001**
	1500	**0.984**	**0.981**	-2.766	0.135	-20.433	**1.69E-07**	1.935	0.303	6.379	**3.75E-04**
KH311	500	0.692	0.648	-0.615	0.155	-3.963	**0.005**	0.235	0.348	0.676	0.520
	1000	**0.728**	0.689	-0.876	0.202	-4.331	**0.003**	0.665	0.453	1.467	0.186
	1500	**0.708**	0.667	-0.818	0.198	-4.125	**0.004**	0.218	0.445	0.490	0.639
rec30	500	**0.966**	**0.961**	-1.182	0.084	-14.055	**2.19E-06**	-1.764	0.188	-9.362	**3.30E-05**
	1000	**0.952**	**0.945**	-1.234	0.105	-11.738	**7.37E-06**	-1.871	0.236	-7.939	**9.57E-05**
	1500	**0.988**	**0.986**	-1.381	0.058	-23.793	**5.89E-08**	-1.532	0.130	-11.776	**7.21E-06**
UVS78	500	**0.834**	**0.811**	-0.411	0.069	-5.935	**0.001**	-0.175	0.155	-1.130	0.296
	1000	**0.831**	**0.807**	-0.439	0.075	-5.868	**0.001**	-0.274	0.168	-1.634	0.146
	1500	**0.903**	**0.889**	-0.536	0.066	-8.072	**8.61E-05**	-0.272	0.149	-1.830	0.110
Space exposed											
R1 (MgF_2)	500	0.378	0.289	-0.227	0.110	-2.061	0.078	-0.923	0.246	-3.746	**0.007**
	1000	**0.717**	0.676	-0.189	0.045	-4.208	**0.004**	-0.893	0.101	-8.857	**4.73E-05**
	1500	0.342	0.248	-0.184	0.097	-1.906	0.098	-0.845	0.216	-3.905	**0.006**
R1 (SiO_2)	dark	**0.863**	**0.844**	-0.193	0.029	-6.652	**2.90E-04**	0.322	0.065	4.948	**0.002**
	500	**0.807**	**0.779**	-0.589	0.109	-5.408	**0.001**	-0.289	0.244	-1.184	0.275
	1000	0.435	0.354	-0.343	0.148	-2.320	0.053	-0.684	0.331	-2.067	0.078
	1500	0.307	0.208	-0.273	0.155	-1.760	0.122	-0.770	0.348	-2.215	0.062
KH311	dark	0.338	0.244	-0.036	0.019	-1.891	0.101	-0.568	0.042	-13.464	**2.93E-06**
	500	**0.710**	0.668	-0.500	0.121	-4.138	**0.004**	-0.192	0.271	-0.708	0.502
	1000	0.536	0.470	-0.078	0.027	-2.846	**0.025**	-0.271	0.062	-4.397	**0.003**
	1500	**0.702**	0.659	-0.132	0.033	-4.060	**0.005**	-0.049	0.073	-0.676	0.520
rec30	dark	**0.731**	0.693	-0.252	0.058	-4.365	**0.003**	-1.418	0.129	-10.980	**1.15E-05**
	500	0.554	0.490	-0.334	0.114	-2.946	**0.022**	-3.152	0.254	-12.395	**5.11E-06**
	1000	0.651	0.602	-0.238	0.066	-3.617	**0.009**	-2.020	0.148	-13.673	**2.64E-06**
	1500	**0.914**	**0.901**	-0.263	0.031	-8.602	**5.72E-05**	-1.594	0.068	-23.283	**6.84E-08**
UVS78	dark	0.104	-0.025	-0.014	0.016	-0.899	0.398	-0.543	0.036	-15.030	**1.39E-06**
	500	**0.906**	**0.891**	-2.218	0.291	-7.626	**2.65E-04**	-0.666	0.683	-0.975	0.367
	1000	**0.912**	**0.900**	-2.097	0.246	-8.525	**6.06E-05**	1.428	0.551	2.592	**0.036**
	1500	**0.919**	**0.908**	-2.218	0.249	-8.924	**4.51E-05**	1.768	0.557	3.175	**0.016**

Number of values for each condition was 9 (n =9), except the space exposed 500 μm thick UVS78 (n = 8). For regression coefficient (slope), H0 (null) hypothesis is that the regression coefficient is zero, and H1 hypothesis is that the regression coefficient (slope) is not zero. For Y-intercept, H0 (null) hypothesis is that the Y-intercept is zero, and H1 hypothesis is that the Y-intercept is not zero. R^2 values larger than 0.7 are considered significant and indicated in red. The *p* values smaller than 0.05 are also indicated in red.

Supplementary Table 6

Comparison of slope and Y-intercept of regression lines of the survival data of* Deinococcus radiodurans *wild type R1 and DNA-repair-gene-mutant strains.

1st strain			2nd strain			Slope					Y-intercept				
Strain	expose	thickness (μm)	Strain	expose	thickness (μm)	difference	*t* value	*p* value	Rejection of H0 hypothesis (no multiple correction)	Multiple correction: Rejection of H0 hypothesis (Benjamini & Hochberg correction)	difference	*t* value	*p* value	Rejection of H0 hypothesis (no multiple correction)	Multiple correction: Rejection of H0 hypothesis (Benjamini & Hochberg correction)
R1	ISS-Cabin	500	R1	Ground	500	-1.813	4.618	3.99E-04	reject	reject	0.875	0.995	0.337		
		1000			1000	-2.530	13.361	2.33E-09	reject	reject	2.825	6.658	1.08E-05	reject	reject
		1500			1500	-2.567	17.621	5.94E-11	reject	reject	2.780	8.518	6.55E-07	reject	reject
	ISS-Cabin	1000		Space (MgF_2)	Dark	-2.662	14.893	5.59E-10	reject	reject	2.009	5.016	1.89E-04	reject	reject
		500			500	-1.517	3.777	0.002	reject		0.544	0.604	0.555		
		1000			1000	-2.666	14.647	6.97E-10	reject	reject	3.224	7.907	1.57E-06	reject	reject
		1500			1500	-2.582	15.527	3.22E-10	reject	reject	2.780	7.461	3.05E-06	reject	reject
	Space (MgF_2)	Dark		Ground	1000	0.131	1.754	0.101			0.817	4.863	2.51E-04	reject	reject
		500			500	-0.296	2.273	0.039	reject		0.331	1.135	0.275		
		1000			1000	0.135	1.643	0.123			-0.399	2.161	0.048	reject	
		1500			1500	0.015	0.132	0.897			6.69E-04	0.003	0.998		
	Space (SiO_2)	500		Space (MgF_2)	500	-0.363	2.343	0.034	reject		0.634	1.827	0.089		
		1000			1000	-0.153	0.993	0.338			0.209	0.604	0.555		
		1500			1500	-0.089	0.488	0.633			0.075	0.182	0.858		
	Space (MgF_2)	Dark		Space (MgF_2)	1000	-0.004	0.074	0.942			1.216	10.125	8.013E-08	reject	reject
	Space (MgF_2)	Dark		Space (SiO_2)	1000	0.149	0.992	0.338			1.007	2.984	0.010	reject	reject
Rec30	ISS-Cabin	500	Rec30	Ground	500	-0.717	3.126	0.007	reject		-0.102	0.199	0.845		
		1000			1000	-1.127	10.147	7.79E-08	reject	reject	0.338	1.359	0.196		
		1500			1500	-1.156	17.513	6.45E-11	reject	reject	0.321	2.168	0.048	reject	
	ISS-Cabin	1000		Space (MgF_2)	Dark	-0.983	8.196	1.03E-06	reject	reject	-0.453	1.685	0.114		
		500			500	-0.847	6.000	3.26E-05	reject	reject	1.389	4.387	0.001	reject	reject
		1000			1000	-0.996	8.024	1.32E-06	reject	reject	0.149	0.536	0.600		
		1500			1500	-1.118	17.047	9.27E-11	reject	reject	0.063	0.426	0.677		
	Space (MgF_2)	Dark		Ground	1000	-0.144	0.046	0.964			0.791	5.210	1.32E-04	reject	reject
		500			500	0.130	0.539	0.599			-1.491	2.751	0.016	reject	reject
		1000			1000	-0.131	1.745	0.103			0.189	1.126	0.279		
		1500			1500	-0.038	0.870	0.399			0.258	2.628	0.020	reject	reject
	Space (MgF_2)	Dark		Space (MgF_2)	1000	-0.013	0.149	0.883			0.602	3.067	0.008	reject	reject
UVS78	ISS-Cabin	500	UVS78	Ground	500	-0.229	3.165	6.89E-03	reject		-0.069	0.426	0.677		
		1000			1000	-0.179	2.223	0.043	reject		-0.236	1.306	0.213		
		1500			1500	-0.397	5.389	9.55E-05	reject	reject	0.121	0.734	0.475		
	ISS-Cabin	1000		Space (MgF_2)	Dark	-0.425	5.548	7.18E-05	reject	reject	0.268	1.563	0.140		
		500			500	1.807	6.086	3.87E-05	reject	reject	0.416	0.630	0.539		
		1000			1000	1.657	6.446	1.53E-05	reject	reject	-1.703	2.955	0.010	reject	reject
		1500			1500	1.683	6.541	1.31E-05	reject	reject	-2.040	3.539	0.003	reject	reject
	Space (MgF_2)	Dark		Ground	1000	0.246	7.253	4.20E-06	reject	reject	-0.504	6.640	1.11E-05	reject	reject
		500			500	-2.036	7.072	8.39E-06	reject	reject	-0.485	0.844	0.414		
		1000			1000	-1.837	7.412	3.29E-06	reject	reject	1.467	2.642	0.019	reject	reject
		1500			1500	-2.080	8.299	8.91E-07	reject	reject	2.162	3.849	0.002	reject	reject
	Space (MgF_2)	Dark		Space (MgF_2)	1000	2.082	8.4482	7.23E-07	reject	reject	-1.971	3.568	0.003	reject	reject

KH311	ISS-Cabin	500	KH311	Ground	500	-0.559	3.498	0.004	reject	reject	0.316	0.882	0.393		
		1000			1000	-0.841	4.119	0.001	reject	reject	0.743	1.625	0.127		
		1500			1500	-0.839	4.226	0.001	reject	reject	0.502	1.129	0.278		
	ISS-Cabin	1000		Space (MgF_2)	Dark	-0.840	4.137	0.001	reject	reject	1.233	2.710	0.017	reject	reject
		500			500	-0.114	0.581	0.571			0.427	0.969	0.349		
		1000			1000	-0.798	3.909	0.002	reject	reject	0.935	2.046	0.060		
		1500			1500	-0.686	3.411	0.004	reject	reject	0.267	0.593	0.563		
	Space (MgF_2)	Dark		Ground	1000	-0.001	0.022	0.983			-0.490	6.455	1.51E-05	reject	reject
		500			500	-0.445	3.504	0.004	reject	reject	-0.111	0.389	0.703		
		1000			1000	-0.043	1.101	0.289			-0.192	2.181	0.047	reject	
		1500			1500	-0.153	4.552	4.52E-04	reject	reject	0.235	3.114	0.008	reject	reject
	Space (MgF_2)	Dark		Space (MgF_2)	1000	0.043	1.277	0.222			-0.298	3.989	0.001	reject	reject
RI	Ground	500	Rec30	Ground	500	0.534	2.378	0.032	reject		0.408	0.811	0.431		
		1000			1000	-0.217	2.794	0.014	reject	reject	1.715	9.850	1.13E-07	reject	reject
		1500			1500	0.026	0.418	0.682			1.007	7.203	4.54E-06	reject	reject
	ISS-Cabin	500		ISS-Cabin	500	-0.562	1.421	0.177			1.386	1.564	0.140		
		1000			1000	-1.621	7.893	1.60E-06	reject	reject	4.202	9.134	2.84E-07	reject	reject
		1500			1500	-1.384	9.401	2.00E-07	reject	reject	3.466	10.504	5.06E-08	reject	reject
	Space (MgF_2)	Dark		Space (MgF_2)	Dark	0.058	0.901	0.383			1.741	12.032	9.04E-09	reject	reject
		500			500	0.108	0.683	0.506			2.230	6.299	1.96E-05	reject	reject
		1000			1000	0.049	0.615	0.549			1.127	6.298	1.96E-05	reject	reject
		1500			1500	0.079	0.779	0.449			0.749	3.302	0.005	reject	reject
RI	Ground	500	UVS78	Ground	500	0.251	3.451	0.004	reject		-1.148	7.029	5.97E-06	reject	reject
		1000			1000	-0.065	0.859	0.405			-0.456	2.704	0.017	reject	
		1500			1500	-0.060	0.962	0.352			-0.452	3.216	0.006	reject	
	ISS-Cabin	500		ISS-Cabin	500	-1.333	3.395	0.004	reject		-0.203	0.231	0.821		
		1000			1000	-2.415	12.609	4.94E-09	reject	reject	2.605	6.069	2.89E-05	reject	reject
		1500			1500	-2.230	14.793	6.11E-10	reject	reject	2.207	6.534	1.33E-05	reject	reject
	Space (MgF_2)	Dark		Space (MgF_2)	Dark	-0.179	5.383	9.65E-05	reject	reject	0.865	11.613	1.42E-08	reject	reject
		500			500	1.992	6.397	2.35E-05	reject	reject	-0.331	0.359	0.725		
		1000			1000	1.907	7.628	2.37E-06	reject	reject	-2.322	4.144	0.001	reject	reject
		1500			1500	2.034	7.629	2.37E-06	reject	reject	-2.613	4.373	0.001	reject	reject
RI	Ground	500	KH311	Ground	500	0.125	1.558	0.141			-1.173	6.548	1.29E-05	reject	reject
		1000			1000	-0.290	3.888	0.002	reject		-0.416	2.488	0.026	reject	
		1500			1500	-0.220	4.026	0.001	reject	reject	-0.561	4.593	4.18E-04	reject	reject
	ISS-Cabin	500		ISS-Cabin	500	-1.129	2.712	0.017	reject		-0.614	0.658	0.521		
		1000			1000	-1.979	7.377	3.47E-06	reject	reject	1.666	2.771	0.015	reject	
		1500			1500	-1.947	8.107	1.17E-06	reject	reject	1.717	3.190	0.007	reject	
	Space (MgF_2)	Dark		Space (MgF_2)	Dark	-0.158	4.554	4.50E-04	reject	reject	0.891	11.475	1.66E-08	reject	reject
		500			500	-0.731	1.676	0.116			-0.731	1.996	0.066		
		1000			1000	-0.623	2.109	0.053			-0.623	5.270	1.18E-04	reject	reject
		1500			1500	-0.795	0.506	0.621			-0.795	3.483	0.004	reject	reject
Rec30	Ground	500	UVS78	Ground	500	-0.283	1.317	0.209			-1.556	3.236	0.006	reject	reject
		1000			1000	0.152	3.284	0.005	reject	reject	-2.171	20.863	6.06E-12	reject	reject
		1500			1500	-0.086	1.922	0.075			-1.459	14.473	8.16E-10	reject	reject
	ISS-Cabin	500		ISS-Cabin	500	-0.771	7.074	5.56E-06	reject	reject	-1.589	6.507	1.39E-05	reject	reject
		1000			1000	-0.795	6.157	2.49E-05	reject	reject	-1.597	5.519	7.56E-05	reject	reject
		1500			1500	-0.846	9.590	1.57E-07	reject	reject	-1.260	6.375	1.72E-05	reject	reject
	Space (MgF_2)	Dark		Space (MgF_2)	Dark	-0.237	3.961	0.001	reject	reject	-0.876	6.530	1.34E-05	reject	reject
		500			500	1.884	6.661	1.56E-05	reject	reject	-2.561	4.069	0.001	reject	reject
		1000			1000	1.858	7.297	3.93E-06	reject	reject	-3.448	6.043	3.02E-05	reject	reject

		1500			1500	1.956	7.808	1.81E-06	reject	reject	-3.363	5.992	3.30E-05	reject	reject
Rec30	Ground	500	KH311	Ground	500	-0.409	1.887	0.080			-1.581	3.251	5.80E-03	reject	reject
		1000			1000	-0.073	1.606	0.131			-2.131	20.950	5.73E-12	reject	reject
		1500			1500	-0.246	7.541	2.70E-06	reject	reject	-1.568	21.489	4.06E-12	reject	reject
	ISS-Cabin	500		ISS-Cabin	500	-0.567	3.215	0.006	reject	reject	-1.999	5.057	1.75E-04	reject	reject
		1000			1000	-0.359	1.573	0.138			-2.536	4.965	2.08E-04	reject	reject
		1500			1500	-0.563	2.722	0.017	reject	reject	-1.750	3.777	2.04E-03	reject	reject
	Space (MgF_2)	Dark		Space (MgF_2)	Dark	-0.216	3.561	0.003	reject	reject	-0.850	6.254	2.11E-05	reject	reject
		500			500	0.166	1.001	0.334			-2.961	7.967	1.44E-06	reject	reject
		1000			1000	-0.160	2.244	0.041	reject		-1.749	10.931	3.07E-08	reject	reject
		1500			1500	-0.130	2.917	0.011	reject	reject	-1.545	15.421	3.53E-10	reject	reject
UVS78	Ground	500	KH311	Ground	500	-0.127	2.869	0.012	reject	reject	-0.025	0.256	0.802		
		1000			1000	-0.225	5.496	7.88E-05	reject	reject	0.040	0.435	0.670		
		1500			1500	-0.159	4.789	2.88E-04	reject	reject	-0.109	1.463	0.166		
	ISS-Cabin	500		ISS-Cabin	500	0.203	1.198	0.251			-0.411	1.079	0.299		
		1000			1000	0.436	2.023	0.063			-0.939	1.943	0.072		
		1500			1500	0.283	1.352	0.198			-0.490	1.045	0.314		
	Space (MgF_2)	Dark		Space (MgF_2)	Dark	0.021	0.853	0.408			0.026	0.466	0.649		
		500			500	-1.718	6.001	3.247E-05	reject	reject	-0.400	0.628	0.541		
		1000			1000	-2.019	8.156	1.09E-06	reject	reject	1.699	3.064	0.008	reject	reject
		1500			1500	-2.086	8.320	8.65E-07	reject	reject	1.818	3.236	0.006	reject	reject

The *t* test of difference of slopes of regression lines and differences of Y-intercept of regression lines were performed according to Ichikawa (1990). In the case of no correction for multiple comparison, $p < 0.05$ was judged to reject null hypotheses (for slope, no difference between slopes of two regression lines (H0), for Y-intercept, no difference between Y-intercepts of two regression lines (H0)). As correction method of multiple comparison, Benjamini & Hochberg correction method (1995) was used. Data for samples with 500 µm thickness, those with 1000 µm (including dark control samples), and those with 1500 µm thickness were separately analyzed for multiple comparison correction. The statistical values resulted in the judgements “rejection of null hypotheses” are shown in red.

2 Supplementary References

Benjamini, Y., Hochberg, Y. (1995) Controlling the false discovery rate: a practical and powerful approach to multiple testing. J. Roy. Statist. Soc. Ser. B57: 289-300. DOI: 10.1111/j.2517-6161.1995.tb02031.x

Ichikawa, K. (1990) Statistics for Bioscience—practical technique and theory. Nanko-do, Tokyo. (in Japanese) ISBN: 978-4-524-22036-6

Lean, J. (1991) Variations in the Sun's radiative output. Rev. Geophys. 29, 505–535. DOI: 10.1029/91RG01895

Rabbow, E., Rettberg, P., Barczyk, S., Bohmeier, M., Parpart, A., Panitz, C. et al. (2015) The astrobiological mission EXPOSE-R on board of the International Space Station. Int. J. Astrobiology, 14, 3–16. DOI: 10.1017/S1473550414000202

Yamagishi, A, Kawaguchi, Y., Hashimoto, H., Yano, H., Imai, E., Kodaira, S. et al. (2018) Environmental data and survival data of *Deinococcus aetherius* from the Exposure Facility of the Japan Experimental Module of the International Space Station obtained by the Tanpopo Mission. Astrobiology 18, 1369–1374. DOI: 10.1089/ast.2017.1751
